# Anal high-grade and late-stage cancer management in low-income setting: a case report

**DOI:** 10.1093/jscr/rjaa423

**Published:** 2020-10-28

**Authors:** Anna Claudia Colangelo, Damiano Pizzol, Mario Antunes

**Affiliations:** Department of Surgery and Organ Transplantation, University of Padua, Padua, Italy; Italian Agency for Development Cooperation, Khartoum, Sudan; Department of Surgery, Central Hospital of Beira, Beira, Mozambique

## Abstract

The burden of cancer is increasing in sub-Saharan Africa due to ageing, common risk factors and population growth. Anal cancer is a human papillomavirus-related rare disease with an incidence rate of 1.8 per 100 000 persons overall with an increasing incidence of by 2% per year in the last three decades. Despite that gold standard management is well described, in low-income countries, there is no possibility for a proper management. We presented a late-stage anal cancer case that reflects the urgent necessity to create the adequate condition for the development of effective oncologic approach including prevention, diagnosis and management.

## INTRODUCTION

Anal cancer (AC) is a rare disease with an incidence rate of 1.8 per 100 000 persons overall with 1.5 per 100 000 in men and 2.0 per 100 000 in women [1]. In the past three decades an increasing incidence of AC by 2% per year has been observed and, in 2008, worldwide have been diagnosed ~27 000 cases [1]. The majority are squamous cell carcinomas, whereas non-squamous include adenocarcinomas and melanomas [1]. AC risk factors include human papillomavirus (HPV) infection, history of sexually transmitted diseases (STDs) or of vulvar or cervical carcinoma, human immunodeficiency virus (HIV) immunosuppression or other forms of immunosuppression, haematological or immunological disorders and smoking [2]. It is estimated that 90% of anal squamous cancers are related to HPV and, in particular, HPV-16 is associated with >75% of these cancers [2]. Interestingly, many studies reported a high rate of anal HPV in HIV-infected women with most reporting prevalence rates >70% [3]. Globally, >4% of all types of cancer cases are associated to HPV and of these ~2% in high-income countries and ~8% in low-income countries, especially in Sub-Saharan Africa [3]. Moreover, HPV-related anogenital cancers are increasing worldwide with an estimation of new cases yearly of 13 000, 27 000 and 27 000 for vaginal, vulvar and AC, respectively [4]. Limited data are available on the impact of HPV and its associated diseases in Mozambique, attesting a high prevalence ranging from 40 to 96% depending on the considered population, and listed the most common and known risk factors, such as multiple sexual partners, reproductive age and other STD, including HIV [5]. A recent study on women aged 30–49 years showed a prevalence of HPV infection of 23.7% and women living with HIV were twice as likely to test positive for HPV as HIV negative women (39.2 vs. 19.9%) [6]. We report a rare case of late stage of AC successfully treated in a low-income setting.

## CASE REPORT

**Figure 1:**
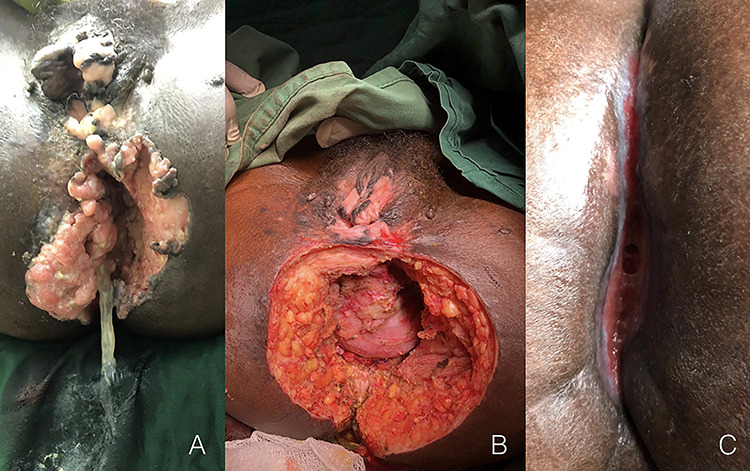
Cantrell syndrome at presentation (**A**), after the surgery procedure (**B**) and after 4 months from treatment (**C**).

A 35-year-old woman was admitted to hospital with a late-stage bleeding and mucous lesion of the anus (Fig. 1A). The patient reported a 5 years of condyloma acuminata in the whole perineal and vulva area that were treated with podophyllin. Despite the treatment and the improvement of some lesions, the patient referred no complete resolution, especially for the anal lesion that worsened during these years with increasing bleeding in the last 8 months. Two years ago, the patient resulted HIV positive and started antiretroviral treatment. At the admission, the patient was anaemic and asthenic with poor general conditions. After transfusion and stabilization of the patient, we performed a colostomy and the biopsy of lesion for the histologic diagnosis that, unfortunately, was not conclusive. We performed a debunking surgery removing the mass as extensive as possible (Fig. 1B). This time, the histologic analysis reported infiltrating epidermal carcinoma. After the surgery, the patient was treated with ampicillin, metronidazole and paracetamol and bandages with hypochlorite were applied daily. The patient was discharged 3 weeks after surgery with the instruction to continue the topic medication. After 4 months from the surgery the patient came for the follow-up visit with the wound almost healed (Fig. 1C). No chemotherapy or radiotherapy was available.

## DISCUSSION

Communicable diseases, such as tuberculosis and HIV, maternal mortality and malaria, are recognized as the main diseases in Africa, whereas cancer is largely under-recognized as a significant health problem. However, the burden of cancer is increasing in sub-Saharan Africa and in 2008, it was estimated that there were 551 221 new cancer cases and 420 978 deaths [7]. Moreover, these numbers are estimated to double in the next 20 years due to ageing, behaviour and growth of the overall population [7]. The HPV-related cervical cancer is the most common cancer diagnosed in women in sub-Saharan Africa, with 75 141 new cases reported per year, and also the leading cause of cancer death at 50 233 deaths and Mozambique has one of the highest incidence rates in the world at >50/100 000 women [7]. Despite these dramatic data, the majority of the low-income settings are unprepared and unable to face the oncologic health issues in a proper way in all aspects including prevention, diagnosis and treatment and this case report reflects this condition of crucial necessity.

In fact, the lack of effective screening programmes and awareness campaign and to contact the health services only after worsening of general health conditions led to a very late stage of malignancy. Moreover, despite the efforts of health workers, and although it would not change the management, it was not possible to perform a diagnosis from the initial bioptic samples, and only after surgery it was possible to have a histological diagnosis. Again, although the dominant aetiology of AC is the HPV infection, it does not confirm the viral presence. Finally, other than the debunking surgery, any other therapy was not available, although the gold standard approach includes chemotherapy plus radiotherapy [2].

In conclusion, although the AC is a rare entity, the viral aetiology is the same of other much more common cancer as cervical, genital and oropharyngeal cancers that are dramatically increasing in the low-income countries both for the spreading of HPV and HIV infection, both for the lengthening of life expectancy. It is mandatory to improve and increase the accessibility of health services, to promote adequate health policies, to strengthen prevention and screening activities and to allocate adequate equipment and human resources.

## ETHICS STATEMENT

Written informed consent was obtained from the patient for publication of this case report and any accompanying images.

## CONFLICT OF INTEREST STATEMENT

None declared.

## FUNDING

None.
